# Inhibition of the Type I Interferon Antiviral Response During Arenavirus Infection

**DOI:** 10.3390/v2112443

**Published:** 2010-11-05

**Authors:** Persephone Borrow, Luis Martínez-Sobrido, Juan Carlos de la Torre

**Affiliations:** 1 Nuffield Department of Clinical Medicine, The Jenner Institute, University of Oxford, Compton, Newbury, Berkshire RG20 7NN, UK; E-Mail: persephone.borrow@jenner.ac.uk; 2 Department of Microbiology and Immunology, University of Rochester, 601 Elmwood Avenue, Rochester, NY 14642, USA; 3 Department of Immunology and Microbial Science, IMM-6, The Scripps Research Institute, 10550 North Torrey Pines Road, La Jolla, CA 92037, USA

**Keywords:** arenavirus, type I interferon, hemorrhagic fever, innate immunity, lymphocytic choriomeningitis virus, nucleoprotein, dendritic cells

## Abstract

Arenaviruses merit interest both as tractable experimental model systems to study acute and persistent viral infections, and as clinically-important human pathogens. Several arenaviruses cause hemorrhagic fever (HF) disease in humans. In addition, evidence indicates that the globally-distributed prototypic arenavirus lymphocytic choriomeningitis virus (LCMV) is a human pathogen of clinical significance in congenital infections, and also poses a great danger to immunosuppressed individuals. Arenavirus persistence and pathogenesis are facilitated by their ability to overcome the host innate immune response. Mammalian hosts have developed both membrane toll-like receptors (TLR) and cytoplasmic pattern recognition receptors (PRRs) that recognize specific pathogen-associated molecular patterns (PAMPs), resulting in activation of the transcription factors IRF3 or IRF7, or both, which together with NF-κB and ATF-2/c-JUN induce production of type I interferon (IFN-I). IFN-I plays a key role in host anti-microbial defense by mediating direct antiviral effects via up-regulation of IFN-I stimulated genes (ISGs), activating dendritic cells (DCs) and natural killer (NK) cells, and promoting the induction of adaptive responses. Accordingly, viruses have developed a plethora of strategies to disrupt the IFN-I mediated antiviral defenses of the host, and the viral gene products responsible for these disruptions are often major virulence determinants. IRF3- and IRF7-dependent induction of host innate immune responses is frequently targeted by viruses. Thus, the arenavirus nucleoprotein (NP) was shown to inhibit the IFN-I response by interfering with the activation of IRF3. This NP anti-IFN activity, together with alterations in the number and function of DCs observed in mice chronically infected with LCMV, likely play an important role in LCMV persistence in its murine host. In this review we will discuss current knowledge about the cellular and molecular mechanisms by which arenaviruses can subvert the host innate immune response and their implications for understanding HF arenaviral disease as well as arenavirus persistence in their natural hosts.

## 1. Arenaviruses as Important Model Systems to Study Virus-host Interactions and as Clinically Relevant Human Pathogens

### 1.1. LCMV Infection of the Mouse: The Rosetta Stone of Virus-host Interactions

Studies using the prototypic arenavirus LCMV have uncovered many key concepts in virology and immunology that have been found subsequently to apply universally to other viral infections of humans, including virus-induced immunopathological disease, T cell-mediated killing of virus-infected cells and major histocompatibility complex (MHC) restriction of T cell responses [1,2]. The outcome of LCMV infection of its natural host, the mouse, varies dramatically depending on the strain, age, immunocompetence and genetic background of the host, as well as the route of infection and the strain and dose of infecting virus [1–3]. This feature of murine LCMV infection provides investigators with a unique model system in which to investigate parameters that critically influence many aspects of virus-host interaction, including those responsible for the heterogeneity of phenotypic manifestations often seen during infections with the same virus.

### 1.2. Arenaviruses and Their Impact on Human Health

**Figure 1.**
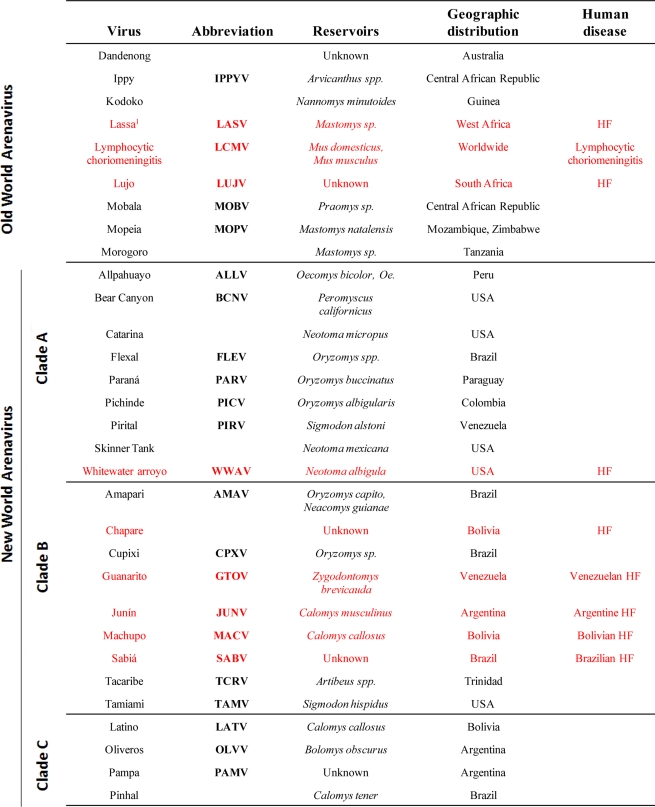
Members of the *Arenaviridae* family. ^1^Arenaviruses known to cause disease in humans are indicated in red.

Arenaviruses cause chronic infections of rodents with a worldwide distribution [3]. Infected rodents move freely in their natural habitat and may invade human dwellings. Humans are infected through mucosal exposure to aerosols, or by direct contact of abraded skin with infectious materials. Several arenaviruses (Figure 1), chiefly Lassa virus (LASV), cause severe hemorrhagic fever (HF) disease in humans, posing a serious public health problem [3–5]. Thus, LASV is estimated to infect several hundred thousand individuals yearly in its endemic regions of West Africa, resulting in a high number of Lassa fever (LF) cases associated with significant mortality and high morbidity. Notably, increased travel to and from endemic regions has led to the importation of LF into non-endemic metropolitan areas around the globe [6–8]. Likewise, the New World (NW) arenavirus Junin virus (JUNV) causes Argentine HF, a severe illness with hemorrhagic and neurological manifestations and a case fatality of 15–30% [5,9,10], whereas the NW Machupo (MACV) and Guanarito (GTOV) arenaviruses emerged as causative agents of HF in Bolivia and Venezuela, respectively [5]. Moreover, compelling evidence indicates that the globally-distributed prototypic arenavirus LCMV is a neglected human pathogen of clinical significance, especially in cases of congenital infection, where it can lead to hydrocephalus, mental retardation and chorioretinitis in infants [11–13]. In addition, LCMV poses a special threat to immunocompromised individuals, as tragically illustrated by recent cases of transplant-associated infections by LCMV with a fatal outcome in the USA [14,15] and Australia [16]. These concerns are aggravated by the lack of licensed vaccines against LASV or LCMV, and current arenavirus therapy being limited to the use of the nucleoside analog ribavirin, which is only partially effective, requires early and intravenous administration for optimal activity and can cause significant side effects.

## 2. Molecular and Cell Biology of Arenaviruses

**Figure 2.**
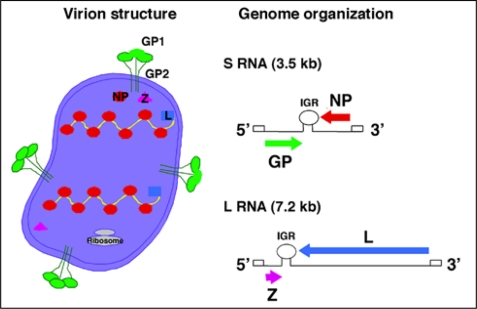
Virion structure and genome organization of arenaviruses. Each genome segment S (*ca.* 3.5 kb) and L (*ca.* 7.5 kb) uses an ambisense coding strategy to direct the synthesis of two different viral polypeptides: S encodes GP and NP, and L encodes the L polymerase and Z. Arenavirus particles are spherical to pleomorphic and contain a number of electron-dense structures thought to be ribosomes that give the particles a “sandy” look under an electron microscope.

Arenaviruses are enveloped viruses with a bi-segmented negative stranded RNA genome and a life-cycle restricted to the cell cytoplasm [3,17]. Each genomic RNA segment, L (*ca.* 7.3 kb) and S (*ca.* 3.5 kb), uses an ambisense coding strategy to direct the synthesis of two polypeptides that are encoded in opposite orientations, separated by a non-coding intergenic region (IGR) with a predicted stable hairpin folding structure (Figure 2). The S RNA encodes the viral glycoprotein precursor (GPC) and the nucleoprotein (NP). GPC is post-translationally cleaved by the cellular site 1 protease (S1P), also known as pro-protein convertase (PC) subtilisin-kexin-isozyme-1 (SKI-1), to yield the two mature virion glycoproteins GP1 and GP2. The L RNA encodes the viral RNA-dependent RNA polymerase (RdRp, or L polymerase), and the small (11 kDa) really interesting new gene (RING) finger protein Z.

The NP and L mRNAs are transcribed into a genomic-complementary mRNA, whereas the GPC and Z coding regions are not translated directly from genomic RNA, but rather from genomic-sense mRNAs that are transcribed using the corresponding antigenome RNA species as templates, which also function as replicative intermediates. The IGR acts as a bona fide transcription termination signal for the virus polymerase [18,19].

Trimers of GP1/GP2 associate via ionic interactions to form the spikes that decorate the virus surface (Figure 2). GP1 is located at the top of the spike and mediates virus interaction with host cell surface receptors. The cellular receptor for LCMV and LASV is α-dystroglycan (α-DG) [20,21]. Upon initial attachment to the target cell, LCMV virions are taken up in smooth-walled vesicles, which are not associated with clathrin [22]. In contrast, the NW arenaviruses GTOV, JUNV, MACV and Sabia (SABV) can use human transferrin receptor 1 as a cellular receptor [23] and clathrin-dependent endocytosis has been reported for JUNV [24]. Fusion between the viral and cell membranes is triggered by the acidic environment of the late endosome, which is thought to trigger conformational changes in the arenavirus GP [25–27], exposing a fusogenic peptide that mediates fusion of the virion and host cell membranes [28,29].

As with many other enveloped negative stranded (NS) RNA viruses, formation and cell release (budding) of arenavirus infectious progeny requires that assembled viral ribonucleoproteins (RNPs) associate at the cell surface with membranes that are enriched in viral GPs. For many enveloped NS RNA viruses this association and subsequent viral budding is mediated by a matrix (M) protein that acts as a bridge between the RNP and GP. We [30] and others [31,32] have shown that Z is the main driving force for arenavirus budding and that this process is mediated by the Z proline-rich late domain motifs (PTAP and PPPY) known to control budding of several other viruses via interaction with specific host cell proteins [33]. The view of Z as the arenavirus counterpart of the M protein found in many other NS RNA viruses is consistent with ultrastructural data on arenavirus virions determined by cryo-electron microscopy [34], as well as genetic and biochemical evidence of a GP-Z association [35], and the ability of Z to inhibit RNA synthesis by the arenavirus polymerase [36].

### Arenavirus Reverse Genetics

The inability to genetically manipulate the arenavirus genome has hampered studies aimed at understanding its molecular and cell biology of arenaviruses, as well as their pathogenesis. The development of reverse genetics systems for several arenaviruses including LCMV has facilitated the investigation of the *cis*-acting sequences and *trans*-acting factors that control arenavirus replication and gene expression, as well as assembly and budding [37–40]. Moreover, the ability to rescue infectious arenaviruses including LCMV [41,42], JUNV [43] and Pichinde virus (PICV) [44] entirely from cloned complementary DNAs (cDNAs) has provided investigators with new avenues to start examining the relationship between predetermined mutations within the virus genome and specific phenotypes, which in the case of LCMV can be assessed in the context of virus infection of its natural host the mouse. This development has provided investigators with novel and unique opportunities to investigate arenavirus-host interactions that influence the variable outcome of infection, which can range from virus control by host defenses to severe acute disease and long-term chronic infection associated with subclinical disease. Likewise, these advances in arenavirus genetics have opened up new approaches for the development of novel vaccine strategies and screening methods to identify and evaluate novel anti-arenaviral drugs targeting specific steps of the virus life-cycle.

## 3. Type I IFN Antiviral Response and Innate Immunity

### 3.1. Type I IFNs and Their Roles in the Innate Response

The type I IFNs (IFN-I) are a group of innate cytokines that comprise multiple subtypes of IFNα (13 in humans), IFNβ (only 1 subtype in humans) and other less well-characterized IFNs such as IFNω, IFNε and IFNκ Production of IFN-I can be triggered very rapidly during virus infection, and these pleiotropic cytokines both combat virus replication directly and also induce and regulate the activation of cells involved in the innate and adaptive responses of the host to the infection. IFN-I thus play a key role in host defense, particularly in the early stages of infection. IFN-I mediate their diverse activities by binding to a broadly-expressed cell-surface receptor, the heterodimeric IFNα/β receptor (IFNAR), an interaction that triggers intracellular signaling cascades that result in up-regulation of the expression of multiple IFN-stimulated genes (ISGs). IFN-I are distinguished from the other members of the IFN family, type II IFN (IFNγ) and type III IFNs (of which there are three subtypes, also called IL-28A/B and IL-29), on the basis of their structure, cell surface receptor usage, the intracellular signaling pathways via which they trigger ISG expression and the pattern of ISGs they up-regulate. Further, although type I and III IFNs can be produced by many different cell types, type II IFN is primarily produced by activated NK cells and T cells (hence is termed lymphocyte IFN).

IFN-I were originally described as factors secreted from virus-exposed cells that rendered other cells resistant to virus infection [45]. Their importance in combating virus infections *in vivo* was subsequently demonstrated in experiments using antibodies that block IFN-I activity or mice that lack the IFN-I receptor (IFNAR knock-out mice), where control of infections with a diverse range of RNA and DNA viruses was found to be impaired [46–50]. Likewise humans who have deleterious mutations in components of the signaling pathways involved in IFN-I induction or their downstream effector molecules are highly susceptible to viral disease, which is thought to be largely due to impairment of IFN-I-mediated control of infection [51–54]. Consistent with the key antiviral roles played by IFN-I, many viruses possess strategies for reducing IFN-I induction, impairing IFN-I activity, and resisting control by IFN-induced antiviral factors [55,56].

#### 3.1.1. Cellular Sources of IFN-I Production

Production of IFN-I is one of the most rapidly-induced host immune responses—it can be up-regulated within hours of virus infection. Most cells have the capacity to produce IFN-I when they become infected with viruses. IFN-I production in infected cells is triggered by the interaction of viral components with host cellular PRRs, which activates signaling pathways leading to IFN-I induction [57]. The IFN-I secreted by infected cells acts in an autocrine fashion, via interaction with its receptor, enhancing IFN-I production and promoting control of virus replication in the IFN-producing cell, and also in a paracrine fashion to facilitate the establishment of an antiviral state in surrounding cells. IFN-I can also modulate the activity of immune system cells in the vicinity of the infection site. Specialized hematopoietic cells, principally plasmacytoid dendritic cells (pDCs), produce the higher levels of IFN-I and other proinflammatory cytokines in response to viral infections [58,59]. Importantly, pDCs can be triggered to produce IFN-I in response to recognition of pathogen components without themselves becoming infected, as they possess unique PRR-coupled signaling pathways for IFN-I induction [57,60]. This enables high levels of IFN-I to be produced in response to infection with viruses that are not pDC-tropic, and also provides a mechanism for generating IFN-I during infections with viruses that impair IFN-I production in the cells they infect. The high levels of IFN-I produced by pDCs induce their maturation, and importantly, act in a paracrine fashion to induce a widespread antiviral state and to activate other cell types involved in the innate and adaptive response [61].

#### 3.1.2. Pleiotropic Effects of IFN-I

Binding of IFN-I to their receptor initiates signaling cascades that result in the induction of hundreds of ISGs [62]. IFN-I-receptor binding activates two members of the Janus tyrosine kinase family, Janus kinase 1 (JAK1) and tyrosine kinase 2 (Tyk2), which in turn activate, by phosphorylation, signal transducer and activator of transcription (STAT) factors STAT1 and STAT2. As a consequence of phosphorylation, STAT1 and STAT2 heterodimerize, translocate to the nucleus and associate with interferon regulatory factor (IRF) 9 to form the IFN-stimulated gene factor-3 (ISGF3) complex. The ISGF3 complex binds specific DNA sequences containing IFN-stimulated response elements (ISREs), promoting the transcription of ISGs.

The pattern of ISG induction is dependent on the quantity and species of IFN-I involved and the type of the responding host cell [63]. A number of ISGs encode PRRs, downstream signaling molecules or transcription factors that are involved in amplifying IFN-I induction—hence IFN-I up-regulates its own production in a positive feedback fashion [64]. For example, one of the ISGs is IRF7, which stimulates production of IFNα, amplifying the IFN-I response. The products of other ISGs mediate direct antiviral effects, acting to combat viral replication at multiple different stages of the viral life-cycle. Examples include ISG15 (a ubiquitin homologue), ribonuclease L (RNase L), protein kinase R (PKR) [65,66], the 2′,5′-oligoadenylate synthetases (2–5OAS) [67], Mx1 and other GTPases [68–71], deaminases such as double-stranded (ds) RNA-specific adenosine deaminase (ADAR1) and apolipoprotein B mRNA editing enzyme, catalytic polypeptide-like (APOBEC)s, ISG20 (an exonuclease) and tripartite motif proteins such as TRIM5α [72]. Some of ISGs have broad activity against multiple viruses (e.g., the 2–5OAS/RNase L and PKR pathways), whilst others target specific classes of viruses (e.g., APOBEC3G combats infection with certain retroviruses). Together, these factors induce a potent antiviral state in IFN-stimulated cells. However, only a relatively small proportion of ISGs are known to have direct antiviral activity. Several ISGs regulate cell growth and survival, with some ISGs indirectly contributing to control of virus replication by stimulating infected cells to undergo apoptosis [73]. Another class of ISGs mediate immunomodulatory effects, enabling IFN-I to activate and regulate the functions of cells involved in both the innate and the adaptive response. Cell subsets whose activity is modulated by IFN-I include natural killer (NK) cells, NKT cells, macrophages and DCs. IFN-I can also enhance the cytokine and chemokine production and the cytolytic activity of NK and NKT cells and macrophages [74]. They also up-regulate Fc-receptor expression on these cells so that their functions are more readily triggered by antibody binding [75].

IFN-I not only induce the maturation of pDCs and enhance their production of cytokines and chemokines, but also play an important role in the activation and maturation of conventional DCs (cDCs) [76]. They enhance cytokine and chemokine production by cDCs including production of interleukin (IL)-15, which has important effects on the proliferation and homeostasis of NK cells and memory CD8+ T cells. As IFN-I drive DC maturation they induce expression of the chemokine receptor CCR7, which promotes DC recruitment into lymph nodes [77]. IFN-I also increase the expression of MHC and co-stimulatory molecules on DCs and promote antigen presentation and cross-priming of CD8+ T cell responses [78]. In addition to enhancing the capacity of DCs to stimulate adaptive responses they also modulate the nature of the adaptive response induced, biasing it in a Th1-direction appropriate for mediating control of virus infections [79,80]. IFN-I further stimulate the adaptive response by acting directly on T and B cells: they enhance CD8+ T cell responses [81,82] and increase antibody production by B cells [83–85].

Immune activation can cause immunopathology if not carefully controlled. Therefore, in addition to promoting the activation of innate and adaptive responses, IFN-I also have important negative immunoregulatory effects. These effects are sometimes mediated indirectly, e.g., IFN-I induce expression of tumor necrosis factor (TNF)-related apoptosis inducing ligand (TRAIL) on NK cells, which enables them to mediate lysis of immature DCs so that the triggering of adaptive responses is temporally limited [86]. The effects can also be mediated directly, e.g., IFN-I induce production of IFNγ by NK cells, with changes in transcription factor expression in NK cells that prevent sustained over-expression of IFNγ [87]. The type and magnitude of the response of a particular cell type to IFN-I is influenced by the differentiation and activation state of the cell and the other signals it concurrently receives [64]. Thus although IFN-I promote the proliferation and enhance the functions of antigen-specific T cells during an immune response, they simultaneously limit the expansion of non-specific T cells that are not receiving stimulatory signals via the T cell receptor [88,89].

#### 3.1.3. Down-regulation of IFN-I Production

Notably, IFN-I production is itself tightly regulated. There is a basal level of constitutive IFN-I production in the healthy, uninfected state, which confers some resistance to viral infection on cells throughout the body and helps to protect against development of tumors. This constitutive IFN-I production keeps cells in a state of “low-level alert” from which they can be rapidly triggered to produce higher levels of IFN-I in response to infection. However over-production of IFN-I in either the short or the long term can have pathological consequences [90–92]. There are thus a number of negative-feedback mechanisms via which IFN-I production can be down-modulated. Notably, pDCs express a variety of receptors that when ligated down-regulate signaling via PRRs to reduce IFN-I induction [60]. Ligands for these receptors are up-regulated by IFN-I, hence providing a negative feedback mechanism for regulation of IFN-I production [93].

In summary, IFN-I have important effector and immunomodulatory roles in the host response to infection. They constitute a rapidly-induced first line of defense against the establishment and spread of infection, mediating direct antiviral activity that forms a key component of the innate effector response. They also activate and enhance the functional capacity of effector cells such as NK cells that participate in the innate response, amplifying their activity. Importantly, IFN-I also form a critical link between the innate response to infection and the adaptive response, helping to activate and modulate the functions of DCs and other antigen-presenting cells and also acting directly on T and B cells to promote the induction of a robust antiviral response. It is thus not surprising that defects in the IFN-I response have such a profound impact on the ability of the host to control virus infections, and that so many viruses possess strategies for impairing and/or evading this response.

### 3.2. Pathways Leading to IFN-I Induction

Pathogen recognition by the innate immune system is mediated by germline-encoded PRRs that detect components of foreign micro-organisms termed pathogen-associated molecular patterns (PAMPs) [94]. There are three broad categories of PRRs: (1) secreted factors e.g., collectins and ficolins, which bind to pathogens and mediate complement activation and opsonization, (2) transmembrane PRRs e.g., Toll-like receptors (TLRs) and C-type lectin receptors, and (3) cytosolic PRRs, such as retinoic acid-inducible gene I (RIG-I)-like receptors (RLRs), Nod-like receptors (NLRs) and cytoplasmic DNA sensors [95]. TLRs and C-type lectin receptors are predominantly expressed on the plasma membrane or within intracellular compartments (such as endosomes and lysosomes) of cell types involved in the immune response, and on certain non-immune cells located at common sites of pathogen entry, e.g., epithelial cells [96,97]. By contrast, RLRs, NLRs and cytoplasmic DNA sensors are expressed in the cytoplasm of most cell types [98–100]. PAMP recognition by TLRs, C-type lectin receptors, RLRs and cytoplasmic DNA sensors leads to the activation of signaling pathways that culminate in the activation of transcription factors such as IRFs, nuclear factor (NF)-κB and activating transcription factor (ATF) 2/cJun that translocate into the nucleus and activate the transcription of inflammatory cytokines or IFN-I, or both [57,96,97,99–104]. NLRs respond to PAMPs and other signals including cellular stress by inducing inflammatory responses including production of IL-1β [96,98,100,103].

#### 3.2.1. TLR-dependent Pathways for IFN-I Induction

**Figure 3.**
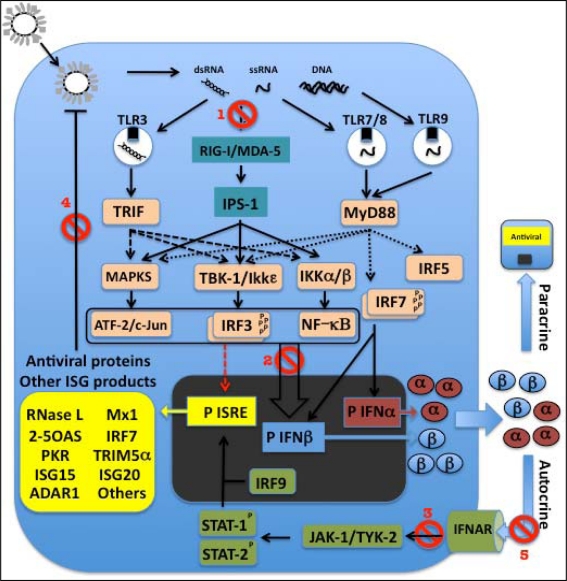
TLR-dependent and independent IFN-I induction, signaling and action. PAMP recognition by PRRs including TLR3 and RIG-I/MDA-5 leads to activation of transcription factors involved in activation of the IFNβ promoter (P). IRF3 is able to activate initial transcription of ISRE promoters by itself (red arrow). Following synthesis, IFNβ is secreted and binds to its receptor to activate the expression of ISGs via the JAK/STAT pathway. ISGs encode proteins with antiviral activity to control viral infection and other factors including IRF7, which leads to production of IFNα, amplifying the IFN-I response. In pDCs, ligation of TLR 7/8 or 9 can also lead to up-regulation of IFN-I production via IRF-7 (which is constitutively expressed in these cells). Different viral mechanisms counteract the IFN-I response at different steps: (1) access of viral PAMPs to PRRs involved in INF-I induction, (2) IFN-I induction, (3) IFN-I signaling, (4) activity of antiviral products, and (5) IFN-I-receptor binding. For more details, see text.

To date, 10 functional TLRs have been identified in humans (TLR1–10) and 12 in mice (TLR1–9 and 11–13) [105]. TLRs are type I transmembrane glycoproteins that have an extracellular domain containing leucine-rich repeats, which mediates recognition of PAMPs; a transmembrane domain; and a cytoplasmic Toll/IL-1 receptor (IL-1R) homology (TIR) domain, which interacts with downstream signaling molecules [106]. TLRs can be broadly divided into two subgroups on the basis of their cellular localization and the PAMPs they recognize. The first group comprises TLRs 1, 2, 4, 5, 6 and 11, which are expressed on the cell surface and mainly recognize microbial membrane components such as lipids, lipoproteins and proteins. For example, TLR4 (together with MD2) binds bacterial lipopolysaccharide (LPS); TLRs 1, 2 and 6 are involved in the recognition of lipopeptides derived from bacteria, fungi parasites and viruses; TLR5 recognizes bacterial flagellin; and TLRs 2 and 4 have been shown to recognize certain viral glycoproteins [57,97,107]. The second group comprises TLRs 3, 7, 8 and 9, which are expressed in intracellular vesicles including endosomes, lysosomes and the endoplasmic reticulum (ER) (oriented with their extracellular domains in the vesicle interior). UNC93B1, a transmembrane protein localized in the ER, plays a key role in transport of the intracellular TLRs to these vesicles [108]. This group of TLRs recognizes microbial nucleic acids derived from pathogens taken up by receptor-mediated endocytosis, phagocytosis or nonspecific fluid-phase endocytosis, or transferred from the cytoplasm into intracellular vesicles by the process of autophagy. TLR3 recognizes dsRNAs including the genomic RNA of reoviruses, the synthetic dsRNA polyinosinic-polycytidylic acid (polyI:C), and in some cases dsRNAs generated during the replication of single-stranded (ss)RNA viruses. TLR7 and human TLR8 recognize ssRNA derived from RNA viruses such as influenza, vesicular stomatitis virus and human immunodeficiency virus (HIV) and RNA species from certain bacteria, plus imidazoquinoline derivatives e.g., resiquimod (R848) (Figure 3). TLR9 recognizes DNA with unmethylated 2′-deoxyribo (cytidine-phosphate-guanosine) (CpG) motifs, which are not commonly found in mammalian DNA, and are usually methylated, but are frequently present in an unmethylated state in bacterial and viral DNA [57,97,104,107].

Engagement of TLRs with their ligands results in triggering of intracellular signaling pathways that culminate in activation of different types of response. All TLRs except TLR3 can associate with the adaptor molecule MyD88, signaling via which leads to activation of NF-κB, mitogen-activated protein kinases (MAPKs) and IRF5 and induction of the expression of inflammatory cytokines. TLR3, and also TLR4, associate with the adaptor TIR-domain-containing adapter-inducing IFNβ (TRIF), which activates different signaling pathways that lead not only to activation of NF-κB and MAPKs and induction of inflammatory cytokine production, but also to activation of IRF3 and induction of IFN-I. In pDCs, ligation of TLR7 or 9 results in activation of a unique MyD88-dependent signaling pathway, which results in activation of NF-κB and IRF5, leading to production of inflammatory cytokines, and also to the activation of IRF7, resulting in IFN-I induction. There are thus two main TLR-dependent pathways for IFN-I induction during virus infections: a pathway involving TLR3-triggered, TRIF-dependent activation of IRF3, which can be utilized in TLR3-expressing cell types including macrophages and cDCs; and a pathway involving TLR7 or TLR9-triggered, MyD88-dependent activation of IRF7, which is unique to pDCs [57,97,101,104].

After binding to dsRNA, TLR3 transmits signals via TRIF resulting in recruitment of tumor necrosis factor receptor short form 1A (TNFRSF1A)-associated via death domain (TRADD) and TNFR-associated factors (TRAFs) 3 and 6 [57]. TRADD and TRAF6 activate MAPKs and NFκB, leading to inflammatory cytokine production. TRAF3 activates the kinases TANK-binding kinase 1 (TBK1) and IκB kinase-ε (IKKε) [109,110]. The RING finger-containing E3 ubiquitin ligase Nrdp1 helps to potentiate TBK1 and IKKε activation [111]. Following activation, these kinases in turn phosphorylate and activate IRF3 [112,113]. Activation of IRF3 is regulated by hyperphosphorylation of Ser and Thr residues at its carboxy-terminal domain [114]. After activation, IRF3 then translocates into the nucleus where it induces transcription of IFNβ and IFNα 4 [115]. Following secretion, these IFN-I bind to the IFNAR and stimulate the induction of ISGs including IRF7, which is expressed at only very low levels in most cells [116,117]. IRF7 is also phosphorylated and activated by TBK1 and IKKε [112], which causes it to translocate to the nucleus. Here it amplifies IFN-I induction, stimulating the transcription of multiple IFN-I subtypes [115,118,119]. Robust IFN-I production by macrophages and cDCs thus involves initial IRF3-dependent induction of some subtypes of IFN-I, followed by amplification of IFN-I production via a positive feedback loop involving IRF7 induction [115,118–120].

In pDCs, production of large amounts of IFN-I can be triggered following binding of viral ssRNA or CpG DNA to TLR7 or TLR9 in endosomes. Retention of the TLR-ligand complex within endosomes is critical for efficient IFN-I induction in pDCs: CpG-A DNA, which is retained in endosomes in pDCs (but not cDCs) is thus a strong stimulus for IFN-I production by pDCs, whereas CpG-B DNA, which rapidly traffics into lysosomes, is not [121]. Endosomal ligation of TLR7 or TLR9 in pDCs triggers recruitment of MyD88, which then complexes with members of the IL-1 receptor associated kinase (IRAK) family, IRAK1 and IRAK4, to form the cytoplasmic transductional–transcriptional processor [122]. The activated IRAKs further associate with TRAF3 and TRAF6, IκB kinase-α (IKKα), and IRFs 5 and 7 [110,123,124]. Unlike other cell types, pDCs constitutively express fairly high levels of IRF7 (although it can be further upregulated via IFN-I) [116,117]. IRF7 is phosphorylated by IRAK1 and/or IKKα [124,125] and activated by TRAF6. Osteopontin is also involved in IRF7 activation [122]. Following activation, IRF7 then translocates into the nucleus where it mediates induction of IFN-I [126]. Production of high levels of IFN-I by pDCs in response to TLR7 or 9 ligation is entirely dependent on IRF7—this transcription factor is thus a “master regulator” of systemic production of IFN-I during virus infections [120,126].

#### 3.2.2. TLR-independent Pathways for IFN-I Induction

Viral 5′-triphosphorylated ssRNA and dsRNA, which are thought to be generated during many viral infections, have been shown to be able to activate cytosolic PRR-mediated pathways for induction of IFN-I [127] (Figure 3). The binding of viral ssRNA and dsRNA to RIG-I stimulates its ATPase/helicase activity, resulting in exposure of RIG-I’s N-terminal caspase recruitment domain (CARD). Because CARD domains are involved in protein-protein interactions, activated RIG-I probably recruits other cellular factors, ultimately leading to the activation of the IRF3 kinases [127]. A protein required to mediate RIG-I signals to downstream factors was identified independently by four different groups and given four different names: mitochondrial antiviral signaling protein (MAVS) [128], IFNβ promoter stimulator 1 (IPS-1) [127], virus-induced signaling adaptor (VISA) [129], and CARD adaptor-inducing IFNβ (CARDIF) [130]. This mitochondrial-localized protein interacts with the CARD domain of RIG-I and recruits and activates IKKε/TBK1, the IKKα/β complex, and MAPK, resulting in activation of IRF3, NF-κB, and ATF-2/c-Jun to induce IFNβ [127].

Melanoma differentiation-associated gene 5 (MDA-5), another PRR that is a close relative of RIG-I, contains RNA helicase and CARD domains, and also mediates initiation of the antiviral response. MDA-5 was found to be targeted by paramyxoviruses, resulting in the inhibition of IFN-I production during viral infection [131–133]. RIG-I and MDA-5 are apparently non-redundant and seem to function in parallel, having a degree of virus specificity [134].

### 3.3. Viral Strategies to Counteract Induction of IFN-I Production

In response to the key role of IFN-I in mediating control of virus infections, many viruses have evolved strategies for impairing IFN-I induction, blocking IFN-I activity, or resisting control by IFN-I-induced antiviral defenses [55,56]. Indeed, a single virus often simultaneously employs a number of different mechanisms for evading the host IFN-I response.

Impairment of IFN-I production can provide a virus with protection from all the antiviral effects of IFN-I, including IFN-I induction of an antiviral state in uninfected cells and the antiviral effector mechanisms triggered by IFN-I via its action on the innate and adaptive response. Consequently, viruses have evolved a variety of strategies aimed at impairing IFN-I production. These can be grouped as follows: (1) reducing the number of specialized IFN-producing cells or impairing their functional capacity; (2) limiting access of viral PAMPs to PRRs involved in IFN-I induction; (3) disrupting the intracellular signaling pathways involved in IFN-I induction following PRR ligation; and (4) blocking the positive feedback loop involved in amplification of IFN-I production.

#### 3.3.1. Viral Effects on the Number and Function of Specialized IFN-producing Cells

Although IFN-I can be produced by most cells when they become infected by viruses, some cell types, especially pDCs, are specialized for production of much higher levels of IFN-I during virus infections. One way in which viruses can limit IFN-I production in their hosts is thus to reduce the number of DCs (particularly pDCs) available for IFN-I production. To achieve this, viruses may block DC recruitment to local sites of virus replication. Alternatively, viruses that cause disseminated infections may induce a reduction in systemic DC numbers, as is observed in HIV-I infection [135,136]. Infection and subsequent death of DCs may be one of the mechanisms contributing to the decrease in their numbers during HIV infection, however the proportion of DCs infected with HIV *in vivo* is thought to be low [137], so other mechanisms, including infection-associated DC activation, lymph node homing and subsequent activation-induced cell death may also contribute to DC loss [138,139]. Viruses may not only reduce the number of pDCs available for IFN-I production, but may also impair the functional capacity of the remaining pDCs. Decreased pDC function has been observed in a number of human persistent virus infections, including chronic infection with hepatitis B or C viruses (HBV or HCV) or human T cell leukemia virus type I (HTLV-I) [140–142]. The mechanisms responsible for the impairment in pDC functions in these infections have not been determined, but one possibility is that circulating virions may interact with surface receptors that down-regulate pDC responses to TLR ligation [60,143]. Viral persistence can also result in chronic exposure of pDCs to low levels of IFN-I that may down-regulate pDC functions.

#### 3.3.2. Restricted Access of PRRs to PAMPs

Viruses can also limit access of the PRRs involved in IFN-I induction to viral PAMPs. For example, bovine viral diarrhea virus (BVDV) secretes a glycoprotein that blocks the ability of extracellular dsRNA to activate TLR3 [144]. Moreover, viruses typically replicate in a way that minimizes the production of PAMPs. Thus, many viruses protect the 5′ end of their mRNA from RIG-I recognition by strategies such as capping (e.g., paramyxoviruses) or coupling a protein to it (e.g., picornaviruses). Viruses can also hide their PAMPs from PRRs, e.g., negative strand viruses encapsidate both genomic and antigenomic RNA, whereas positive strand RNA viruses replicate within intracellular membrane vesicles. Likewise, many viruses encode dsRNA binding proteins to sequester dsRNA.

#### 3.3.3. Disruption of Cell Signaling Pathways Leading to IFN-I Induction in Infected Cells

Viruses can limit IFN-I production in the cells they infect by interfering globally with host cell gene expression or protein synthesis, or both. However numerous viruses can specifically disrupt intracellular signaling pathways involved in IFN-I induction following PRR ligation. If viruses target early steps in intracellular signaling cascades this may impair IFN-I production in some but not all of the pathways leading to IFN-I induction in infected cells, for example the NS3/4a protein of HCV cleaves TRIF and impairs IFN-I induction via TLR3 without affecting pathways driven by cytosolic sensors [145]. Likewise, the V protein of most paramyxoviruses inhibits IFN-I induction by MDA-5, but not RIG-I [146]. It is thus perhaps not surprising that many viruses target molecules that are downstream in the signaling cascades. Because IRF3 plays a key role in the induction of the IFN-I response [147,148], it is one of the cellular factors most commonly targeted by both RNA and DNA viruses to inhibit the IFN-I response in infected cells.

#### 3.3.4. Virus Interference with Mechanisms for Amplification of IFN-I Production

Viruses can disrupt the mechanisms leading to amplification of IFN-I production, which also affects downstream antiviral activities of IFN-I. Thus, some poxviruses encode soluble proteins that bind to IFN-I, hence preventing it from binding to the IFNAR. Evidence indicates that almost every step in the signaling cascades that link IFNAR ligation to ISG expression can also be targeted by viruses, with many viruses antagonizing more than one step in the process [55].

## 4. The IFN-I Response during Arenavirus Infections

### 4.1. IFN-I Production in Acute and Chronic LCMV Infections

#### 4.1.1. The IFN-I Response in Acute LCMV Infection

**Figure 4.**
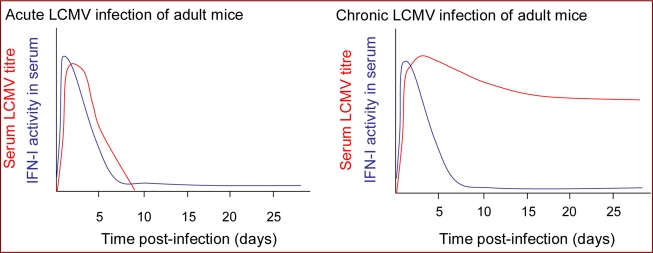
Diagram illustrating the dynamics of viral replication and IFN-I production in adult mice infected with LCMV isolates that induce an acute (left) or a chronic (right) infection.

Acute LCMV infection of mice is associated with rapid induction of a high magnitude burst of IFN-I production [74,149] (Figure 4). IFN-I mRNA expression in the spleen is up-regulated within six hours of infection and peak IFN-I levels in the spleen and circulation are reached within 24 or 48 hours in mice infected with LCMV intravenously or intraperitoneally, respectively, [150–152], and begin to decline well before peak in virus replication, which is typically reached at 3–5 days post-infection [74,151,152]. Notably, the magnitude and kinetics of the IFN-I response induced during the acute phase of infection with LCMV isolates whose replication is subsequently controlled by the host CD8+ T cell response are very similar to those that induce T cell exhaustion and go on to establish a persistent infection [151,152] (Figure 4).

Despite the limited duration of high-level IFN-I production in acute LCMV infection, the early IFN-I response is a critical determinant of the efficiency of control of virus replication. Neutralization of IFN-I activity by administration of anti-IFN antibodies to mice enhances LCMV replication [47]. Likewise LCMV replication is markedly enhanced in mice lacking the IFNAR, and although low-dose infections are eventually controlled (with delayed kinetics) [153], infection with moderate doses of LCMV results in T cell exhaustion and virus persistence [48,49].

#### 4.1.2. Mechanisms by Which IFN-I Mediate Control of LCMV Infection

IFN-I mediates control of LCMV infection via both direct and indirect mechanisms. IFN-I production in LCMV-infected mice is associated with the rapid induction of many ISGs [154,155], at least some of which likely contribute to constraining LCMV multiplication. As yet their specific contributions and mechanisms of actions are not fully understood [155–161], although two recent studies showed that tetherin, expression of which is up-regulated by IFN-I, inhibits the release of arenaviruses including LASV from host cells [162,163], suggesting that this may be among the mechanisms contributing to IFN-I-mediated control of the replication of LCMV and other arenaviruses. IFN-I stimulate the activation and proliferation of NK cells on days 2–3 post-infection, but depletion studies suggest that these innate effector cells do not make an appreciable contribution to control of LCMV infection [164–166]. Therefore, the direct antiviral activity of IFN-I likely accounts for much of their effect on LCMV multiplication during the first few days of an acute LCMV infection.

IFN-I also play an indirect role in control of LCMV multiplication, by contributing to the induction of the virus-specific CD8+ T cell response. They activate DCs [167] and induce them to mediate cross-priming [168] and act directly on CD8+ T cells to promote their clonal expansion and stimulate memory formation [81,169]. Nonetheless, there are also other mechanisms for DC activation in LCMV-infected mice [167] and when mice lacking the IFNAR are infected with low doses of LCMV, virus-specific CD8+ T cells still undergo expansion and are stimulated to produce IFNγ by an IL-12-dependent pathway [153]. IFN-I thus probably have a more important impact on the LCMV-specific CD8+ T cell response via their antiviral activity: whether or not LCMV infection is eventually cleared or instead goes on to persist is crucially dependent on whether the early IFN-I response is able to keep viral titers at levels that do not drive exhaustion of virus-specific CD8+ T cells [170]. If subject to excessive stimulation in the presence of high levels of LCMV replication, CD8+ T cells undergo a process of exhaustion where their functions are impaired and they may be driven to undergo apoptosis; without the activity of these pivotal effector cells the infection cannot be cleared, and LCMV persists [171,172]. Although a virus-specific CD8+ T cell response is induced following low-dose LCMV infection of IFNAR mice, the epitope-specific response of highest avidity (which is most prone to exhaustion [172]) is impaired [153]; and moderate doses of rapidly-replicating LCMV isolates readily exhaust the overall CD8+ T cell response in IFNAR-deficient mice [48,170]. Hence although IFN-I impact on the CD8+ T cell response in LCMV infection in multiple ways, their most critical role is to constrain early virus replication sufficiently to prevent CD8+ T cell exhaustion.

#### 4.1.3. IFN-I Production during Chronic LCMV Infection

Chronic LCMV infection, whether established at birth or initiated following persistence of an LCMV infection acquired in adulthood, is associated with only a modest elevation in IFN-I levels [151,152,173–175]. The ongoing IFN-I production is clearly insufficient to mediate viral clearance; nonetheless it may have some beneficial consequences. The IFN-I production induced in response to acute LCMV infection can inhibit the replication of other viruses [176]; and likewise mice chronically infected with LCMV, despite their relatively muted IFN-I production, do exhibit chronic up-regulation of ISGs [177] and are reported to exhibit resistance to superinfection with certain other viruses [178,179]. The ongoing IFN-I production in mice chronically infected with LCMV from birth also maintains NK cells in a heightened state of activation [174], which may contribute to control of unrelated virus infections and has been shown to confer resistance to tumors [174]. Conversely, chronic up-regulation of IFN-I can also have detrimental consequences. Mice infected with LCMV from birth can fail to gain weight, undergo liver cell necrosis and then die, effects that are dependent on IFN-I [180,181]. Certain mouse strains also develop a glomerulonephritis when persistently infected from birth with LCMV in which IFN-I play a key immunopathological role [182,183]. Chronic IFN-I production can also promote the development of autoimmunity, by activating antigen presenting cells to stimulate rather than tolerise autoreactive cells [184,185].

#### 4.1.4. Cellular Sources of IFN-I Production in LCMV-infected Mice

Despite the important role played by IFN-I in LCMV-host interactions, the cellular sources of IFN-I production in acute and chronic LCMV infection remain incompletely understood. As discussed in Section 3, most cell types are able to produce IFN-I when they are infected by viruses, but some hematopoietic cells, most notably pDCs, which are specialized for high-level IFN-I production during virus infections, can also be stimulated to produce IFN-I following recognition of viral components without themselves being infected. A number of studies have shown that pDCs are rapidly activated to produce IFN-I in acute LCMV infection [152,167,186]. cDCs from LCMV-infected mice also produce IFN-I [187], but they up-regulate IFN-I production with slightly slower kinetics and produce less IFN-I than pDCs [152]. Likewise DC subsets contribute to ongoing low-level IFN-I production in mice chronically infected with LCMV [152]. However although pDCs in LCMV-infected mice do produce IFN-I, high levels of IFN-I are still produced in response to LCMV infection in pDC-depleted animals [188], indicating that other cell type(s) also make an important contribution to the IFN-I response. These other cells may include one or more of the leukocyte populations found in the splenic marginal zone, as IFN-I production in response to LCMV infection is impaired in mice where the normal splenic architecture is disrupted and marginal zone organization is lacking. Notably, cells in the marginal zone constitute the initial sites of LCMV replication in the spleen [189], and LCMV dissemination and replication within this organ at early times post-infection is also impaired by disruption of the marginal zone organization [190]. The marginal zone contains reticuloendothelial cells and leukocyte populations including metallophilic macrophages (MM), marginal zone macrophages (MZM), MZ B cells and cDCs. Depletion of the phagocytic populations (MM, MZM and cDCs) from the marginal zone using clodronate-containing liposomes impaired IFN-I production in response to LCMV infection; and complete re-population, including restoration of MZM, which are reconstituted with the slowest kinetics, was required to reconstitute the normal IFN-I response [150]. However it remains unclear whether the importance of marginal zone structure for the IFN-I response in LCMV-infected mice is due to it serving to locate IFN-producing cells at the site of virus entry to the spleen, and/or whether the marginal zone provides support for intracellular communication and/or transfer of material that is necessary for induction of maximal IFN-I production (perhaps mediated by cells elsewhere). Nonetheless, it appears that several hematopoietic populations, potentially including specialized macrophages as well as pDCs, may be involved in IFN-I production in LCMV-infected mice.

#### 4.1.5. Pathways Involved in IFN-I Induction During LCMV Infection

How IFN-I production is triggered in LCMV-infected mice is also not fully understood. LCMV components that could act as ligands for PRRs involved in IFN-I induction include the viral ssRNA and dsRNAs generated during viral replication, which could be recognized by TLR7 (ssRNA), TLR3 (dsRNA following autophagy or endocytosis of infected cells), as well as RLRs in the cytoplasm of infected cells (e.g., RIG-I, which recognizes ssRNA with 5′ triphosphates/short dsRNAs and MDA-5, which recognizes longer dsRNAs). Recently, it has been shown that upon transfection into IFN-competent cells LCMV genomic RNA is able to induce IFN-I responses by activation of the RNA helicases RIG-I and MDA-5 [191]. However other *in vitro* studies suggest that the single unpaired 5′ ppp-G nucleotide found where the complementary ends of the arenavirus genome anneal to form a dsRNA panhandle is not recognized by RIG-I [192], suggesting that MDA-5 may play a more important role in triggering IFN-I production in LCMV-infected cells. In MyD88-deficient mice the increase in serum IFN-I levels during acute LCMV infection was found to be lower than that in control animals [186], likely reflecting a role for TLR7-triggered MyD88-dependent signaling in IFN-I induction. Consistent with this IFN-I production was also diminished in LCMV-infected TLR7/TLR9-deficient mice [186], although IFN-I production in these animals seemed to be impaired to a lesser extent than in MyD88-deficient mice. Likewise studies in mice deficient in IRAK4, a downstream signaling component of the TLR7/9-MyD88 pathway for IFN-I induction, suggested that there was little defect in early control of LCMV replication in these animals, although their virus-specific T cell response may be impaired [193–195]. There may thus be a role for MyD88-dependent signaling through TLRs in addition to TLR7/9 in IFN-I induction. TLR2, TLR4 and TLR8 do not appear to be involved in IFN-I induction during LCMV infection, as a strong systemic IFN-I response was elicited to LCMV in mice lacking each of these receptors [186]. However induction of inflammatory cytokine and chemokine production in astrocytes and microglial cells in response to LCMV infection *in vitro* was reported to occur by a TLR2 and MyD88-dependent pathway [196], raising the possibility that the LCMV glycoprotein may act as a TLR2 ligand that stimulates production of cytokines other than IFN-I. TLR3 (which triggers IFN-I production in a MyD88-independent, TRIF-dependent manner) does not appear to play a major role in induction of systemic IFN-I production during acute LCMV infection, as innate and adaptive responses are normal in mice lacking TLR3 or TRIF [186,197]. However signaling through cytoplasmic RLRs does appear to be important, as the increase in serum IFN-I levels during acute LCMV infection of IPS-I-deficient mice was found to be lower than in control animals [186]. A further study showed that serum IFN-I levels in both MDA-5- and IPS-I-deficient mice were normal on day 1 post-LCMV infection, but were significantly reduced compared to those in wild-type mice on day 2 post-infection, suggesting that the MDA-5/IPS-I pathway is involved in the later phase of IFN-I induction [191]. Together, these studies suggest that IFN-I induction during acute LCMV infection involves MyD88-dependent signaling through TLR7 and possibly other TLRs, which likely occurs in pDCs and other specialized hematopoietic cells; and also IPS-I-dependent signaling through RLRs, which may occur in hematopoietic and non-hematopoietic cells infected with LCMV. However it cannot be excluded that other IFN-induction pathways may also play a role.

#### 4.1.6. Down-regulation of IFN-I Production in LCMV-infected Mice

As noted above, high-level IFN-I production in acute LCMV infection occurs very transiently, declining prior to the peak in viral replication and remaining low thereafter, even if persistent viral replication continues at moderate-high levels. A number of mechanisms likely contribute to the initial decline in IFN-I production in acute LCMV infection and subsequent low-magnitude response during the ensuing chronic infection; and some or all of these may also account for the relatively low-level IFN-I production in mice chronically infected from birth with LCMV. First, acute LCMV infection of mice is associated with a rapid and (if infection persists) long-lasting decline in the number of pDCs and other hematopoietic cells both in the spleen and elsewhere [151,152,167,198], cell types which are thought to constitute an important source of IFN-I production. Second, there is an impairment in the ability of the remaining DCs to produce IFN-I in response to PRR ligation [151,152]. Third, the LCMV nucleoprotein has been shown to prevent the activation and nuclear translocation of IRF3, which provides a powerful means of impairing IFN-I induction in response to RLR triggering in LCMV infected cells. Together, these strategies for down-regulation of IFN-I production (which are discussed in more detail in Sections 4.2 and 4.3 below) achieve the dual goals of enabling ongoing LCMV replication and minimizing IFN-mediated immunopathological damage, thus facilitating LCMV persistence.

### 4.2. Effects of LCMV on DC Biology

#### 4.2.1. DC Activation

Acute LCMV infection of mice is associated with the rapid activation of both pDCs and cDCs. As discussed earlier, pDCs are triggered to produce IFN-I and other cytokines/chemokines within hours of LCMV infection of mice [152,167,186]. They also undergo phenotypic maturation, up-regulating expression of MHC class I and II and costimulatory molecules [167,186], which enhances their ability to mediate antigen presentation to T cells, although cDCs are much more important in this regard. cDCs also undergo a rapid phenotypic and functional maturation during acute LCMV infection. Expression of MHC class I and II and costimulatory molecules is up-regulated, production of IFN-I and inflammatory cytokines and chemokines is initiated, and both the CD4+ and CD8α+ cDC subsets acquire the ability to stimulate the activation and proliferation of LCMV-specific T cells [167]. As cDCs mature they also up-regulate CCR7 expression and migrate into the T cell areas of the spleen and lymph nodes, positioning themselves to mediate optimal T cell priming [167,199]. Priming of the LCMV-specific CD8+ T cell response requires DCs, and cannot be mediated by other antigen presenting cells such as macrophages or B cells [200]. The CD8α+ cDC subset plays the dominant role in CD8+ T cell priming during LCMV infection, as this subset has the unique capacity to mediate cross-priming [201]. As discussed above, cross-priming is enhanced by IFN-I [168]. Together, different DC subsets thus play a central role in stimulating the activation of both innate and adaptive control of LCMV replication.

DC activation during LCMV infection is probably induced by a number of different stimuli. DCs can be directly stimulated by viral components, e.g., as discussed above, DCs express PRRs including TLR7 (pDCs) and TLR2 (cDCs) that have been implicated in LCMV recognition and the activation of IFN-I (TLR7) or inflammatory cytokine (TLR2) production [186,196]. IFN-I and other cytokines also contribute to their activation. cDC activation is reduced in IFNAR-deficient mice [167], but these cells are still sufficiently activated by other mechanisms to mediate CD8+ T cell priming [153]. In addition to being stimulated by soluble factors, DC activation is also enhanced by cross-talk with other leukocyte populations, including iNKT cells, T cells and B cells [202,203].

In mice chronically infected with LCMV, DCs are sustained in a heightened state of activation, likely due to continuous exposure to activating stimuli [151,152]. Although this may help to activate responses that constrain ongoing virus replication (e.g., IFN-I, which is produced by cell types including DCs, drives up-regulation of ISGs with antiviral activity [177]), as discussed earlier it may also have detrimental consequences, e.g., promoting the development of autoimmune responses [184,185].

#### 4.2.2. DC Loss

The activation of pDCs and cDCs in acute LCMV infection is followed by a rapid decline in their numbers [151,152,167]. DC numbers decline in multiple organs, suggesting that these cells are lost, rather than moving from one location to another [151,152]. If the infection is cleared, DC numbers eventually normalize, but if LCMV persists DC numbers remain depressed [151,152]. Some LCMV isolates are able to infect DCs, which can lead to targeting of these cells for immunopathological destruction by virus-specific CD8+ T cells [189,204]. However a marked decline in DC numbers is also observed in the acute phase of infection with LCMV isolates that do not infect DCs efficiently, indicating that other mechanisms must be involved [38,151,167]. Mature DCs have a short lifespan (∼three days [205]), and are continuously undergoing apoptosis and being replaced. DCs isolated from LCMV-infected mice exhibit an enhanced tendency to undergo apoptosis *ex vivo*, and large numbers of apoptotic DCs are also observed in the lymphoid tissues of mice during acute LCMV infection [167,206]. IFN-I play an important role in driving the increase in the rate of DC apoptosis after LCMV infection, as high levels of DC apoptosis are not observed in LCMV-infected IFNAR-deficient mice [167]. Apoptosis gene expression array analysis of CD8α+ cells (including CD8α+ DCs) from mice acutely infected with LCMV revealed an increase in IL-10 mRNA [206]. IL-10 is known to suppress the induction of antiapoptotic genes in DCs, which can trigger their apoptosis, suggesting a mechanism by which DC loss may be induced in LCMV-infected mice [206].

Importantly, DC loss in uninfected mice is balanced by replacement of these cells from the bone marrow. During LCMV infection, DCs not only undergo apoptosis at an enhanced rate, but their replacement is also impaired due to disruption of hematopoesis. Acute LCMV infection induces a pancytopaenia associated with a decrease in the number of pluripotent and committed progenitor cells in the bone marrow that, like the loss of DCs in the periphery, is also induced by IFN-I [207,208]. Notably, megakaryocytes are among the cell types affected by IFN-I, and LCMV infection can be associated with thrombocytopaenia [208]; likewise a recent study suggested that IFN-I-mediated inhibition of megakaryopoiesis may play an important role in the pathogenesis of the hemorrhagic fever associated with JUNV infection [209]. The hematopoietic precursors affected also include DC precursors, whose development and expansion is inhibited via IFN-I signaling through a STAT2-dependent pathway [198,210]. The low level of ongoing IFN-I production in mice that go on to become chronically infected with LCMV may be sufficient to maintain the suppression of hematopoiesis, accounting for the fact that peripheral DC numbers are never replenished [151,152,198]. It is also possible that destruction of infected DCs or DC precursors, or both, by the residual virus-specific CD8+ T cell response contributes to the failure of normalization of DC numbers in mice that fail to control acute LCMV infection and become persistently infected.

#### 4.2.3. Impairment of DC Functions

Although the reduction in the number of DCs (and likely also other hematopoietic cells involved in IFN-I production) in LCMV-infected mice must contribute to the down-regulation of IFN-I production, this probably does not provide the whole explanation for the rapid shut-off of high level IFN-I production in acute infection or the muted IFN-I response observed during subsequent virus persistence. Importantly, DCs isolated from mice acutely or chronically infected with LCMV also exhibit a selective impairment in their ability to produce IFN-I in response to TLR ligation [151,152]. Unlike the reduction in DC numbers, this impairment in DC functions still occurs in IFNAR-deficient mice, hence it is not mediated by IFN-I [151]. The mechanisms responsible are not fully understood, but may involve signaling through negative regulatory receptors [60].

The abnormalities in DC numbers and function in mice in which LCMV persistence is initiated in adulthood by infection with rapidly-replicating virus isolates that induce CD8+ T cell exhaustion are associated with profound impairments in the ability of these animals to mount effective immune responses to other pathogens. The defect in TLR-stimulated pDC production of IFN-I impairs NK cell activation [151]; and they mount only weak T cell responses following infection with other viruses including influenza, vaccinia and herpes simplex [189,204]. Interestingly, mice in which LCMV persistence is initiated at birth exhibit enhanced NK activity [174] and do not have similarly profound abnormalities in their ability to induce T cell responses to other viruses [211]. The number and IFN-producing capacity of DCs in these animals has not been directly addressed—but their higher level of general immune competence indicates that if there are abnormalities in DC numbers and/or functions in these animals they must be less marked than in mice in which LCMV persistence is initiated in adulthood. The relatively low levels of ongoing IFN-I production in mice persistently infected with LCMV from birth may thus be primarily due to defects in the number and IFN-producing capacity, or both, of non-DC IFN-producing cells, coupled with LCMV’s ability to impair IFN-I production in response to RLR triggering in the cells it infects, a mechanism (discussed below) that also limits IFN-I production during acute LCMV infection and during persistent infections initiated in adulthood.

### 4.3. Molecular Mechanisms of LCMV Inhibition of Induction of IFN-I

Virus-stimulated induction of reporter gene expression mediated by IFNβ and ISRE promoters was found to be inhibited by the NP of LCMV and many other arenaviruses [212], suggesting that the anti-IFN-I activity associated with NP likely interfered with IRF3 activation. Consistent with this possibility, nuclear translocation of IRF3 was impaired in LCMV-infected cells and cells expressing the NP of LCMV [212]. Likewise, IRF3-dependent reporter gene expression was also inhibited by NP expression in transfection-based assays. These findings indicated that the IFN-I- counteracting activity of NP involved an early block in the IRF3 activation pathway, which represented the first example of a viral nucleoprotein with IRF3 inhibitory properties, in contrast to ribonucleoprotein complexes of other negative-stranded RNA viruses, such as measles virus and Vesicular Stomatitis Virus (VSV), which activate IRF3.

#### 4.3.1. Differential Inhibition of IFN-I by Arenavirus NPs

The family *Arenaviridae* consists of one unique genus (Arenavirus) that contains more than 20 recognized virus species that are classified into two distinct groups: OW and NW arenaviruses. This classification was originally established based on serological cross-reactivity but is well supported by recent sequence-based phylogenetic studies. OW arenaviruses constitute a single lineage, while NW arenaviruses segregate into clades A, B, and C (Figure 1).

Studies examining the ability of the NPs of different arenaviruses, including both OW and representative members of the existing NW arenavirus lineages, to inhibit IFN-I induction indicated that in cell-based assays all arenaviral NPs tested, with the exception of Tacaribe virus (TCRV) NP, were capable of inhibiting both transcriptional activity and nuclear translocation of IRF3 to levels comparable to LCMV NP [213]. Notably, a recent report indicated that the Z proteins of several NW arenaviruses, including TCRV, (but not OW arenaviruses such as LCMV), were able to bind to RIG-I and inhibit IFN-I induction via the RIG-I signaling pathway [214]. However unlike the NP-mediated inhibition of IRF3 activity, this more limited impairment of the RIG-I signaling pathway may not be sufficient to completely block IFN-I induction in arenavirus-infected cells. Consistent with this hypothesis, and in contrast to observations with LCMV-infected cells, TCRV-infected A549 cells did not rescue replication of the IFN-sensitive Newcastle Disease Virus (NDV) following induction of the cell antiviral state [213]. Accordingly, treatment of Vero cells with tissue culture supernatants from TCRV-infected A549 cells resulted in restricted multiplication of VSV, indicating the presence of IFN-I in the supernatant of TCRV-infected A549 cells. Together, these findings suggest that TCRV does not fully inhibit production of IFN-I [213].

Arenaviruses cause chronic infections of rodents worldwide. Each arenavirus species is associated with a particular rodent host species, except for TCRV [215]. TCRV has not been isolated from rodents but rather from fruit-eating bats in Trinidad. In contrast to other arenaviruses, TCRV does not appear to be capable of establishing persistence in rodents. Whether the reduced ability of TCRV-NP to counteract induction of IFN-I contributes to this feature remains to be determined.

Phylogenetically, TCRV belongs to clade B of the NW arenaviruses, which also contains several HF arenaviruses (JUNV, MACV, SABV, GTOV) whose NPs counteracted IFN-I induction [213]. The differences between TCRV and the HF MACV and JUNV regarding the inhibitory properties of their respective NPs on the IFN-I system would suggest a possible correlation between the anti-IFN-I activity of NP and the pathogenic potential of the virus. However, several non-pathogenic arenaviruses have been shown to be capable of interfering with the induction of IFN-I to levels comparable to LCMV. As with many other viruses, arenavirus virulence is likely to be a polygenic trait. Therefore, the ability of the viral NP to interfere with induction of the IFN-I system may be a necessary but not sufficient factor in arenavirus virulence and pathogenicity.

#### 4.3.2. Amino Acid Residues Critical for the Anti-IFN-I Activity of LCMV-NP

Viral antagonists of the IFN-I response include structural and nonstructural proteins. Some of these viral anti-IFN-I proteins are dispensable for viral replication in IFN-I-compromised systems, e.g., influenza viruses non-structural protein 1 (NS1) [216–218], whereas others are key components of the viral replication machinery, e.g., Ebola VP35 [219]. In the latter case, mutations affecting the anti-IFN-I activity of the protein may compromise its functions in viral multiplication, thus making it difficult to assign specific functions to particular amino acid residues. The nucleoprotein of arenaviruses plays, together with the viral polymerase (L), a central role in transcription and replication of the viral genome [220]. Additionally the arenavirus NPs are required for promoting generation of infectious virus particles [38]. Mutations designed to affect the anti-IFN-I function of the viral NP may, therefore, affect these other protein functions. TCRV NP does not inhibit induction of IFN-I but is competent in viral multiplication and viral production, suggesting that the roles of the arenavirus NP in RNA synthesis and formation of infectious virus particles could be segregated from the IFN-I-inhibiting activity.

Mutation-function studies using series of both N-and C-terminal deletions in the LCMV NP and cell-based assays to measure IFN-I-mediated transcriptional activation of reporter genes identified two domains within the C-terminus region of NP (residues 370–400 and 500–553) that played a critical role in counteracting the IFN-I response [221]. Subsequent studies using a large collection of single amino acid substitutions to alanine (A), as well as small deletions, within regions 370–400 and 500–553 of LCMV-NP led to the identification of residues 382, 385, and, to a lesser degree, 386, as critically required for the anti-IFN-I activity of NP but dispensable for virus RNA replication and transcription activity, as well as formation of infectious viral particles. The same corresponding residues were also required for the anti-IFN-I activity of LASV NP [221].

The identified motif (DIEG) spanning residues 382–385 is highly conserved between OW and NW arenaviruses, including TCRV NP. Because TCRV-NP has impaired anti-IFN-I activity, it appears that other amino acid residues located outside the DIEG region likely contribute to the anti-IFN-I activity of NPs of other arenaviruses. Consistent with this view, C-terminal deletions of more than five residues in LCMV-NP impaired the anti-IFN-I activity of this NP though its DIEG motif was not altered. Therefore, in addition to this DIEG motif, a specific combination of amino acid residues within the C-terminal part of NP is also required for the anti-IFN-I activity of the viral polypeptide. Futures studies, including structure elucidation, will help to elucidate the contribution of these (and other) specific amino acid residues to NP function [221].

Consistent with results from cell-based assays assessing the anti-IFN-I activity of the NP, a recombinant LCMV, generated via reverse genetics, containing the D382A substitution in NP, rLCMV/NP(D382A), showed impaired virus multiplication in IFN-competent cells, suggesting an attenuation of NP’s anti-IFN-I activity. Likewise, rLCMV/NP(D382A)-infected A549 cells exhibited a reduced ability to rescue the interferon-sensitive NDV, as compared with wild-type LCMV. In addition, treatment of Vero cells with tissue culture supernatants from rLCMV/NP(D382A)-infected A549 cells resulted in the establishment of an antiviral state in Vero cells that inhibited replication of VSV. The induction of the antiviral state was prevented by treatment of the tissue culture supernatant with IFN-I neutralizing antibodies, demonstrating that the antiviral state was triggered by the presence of IFN-I in the tissue culture supernatants of rLCMV/NP(D382A)-infected A549 cells [221].

It should be emphasized that, as observed for many other viruses, arenavirus virulence is likely to be a polygenic trait and therefore the ability of LCMV-NP to counteract the induction of IFN-I may be a necessary but not sufficient factor in arenavirus pathogenesis. The biological characterization of recombinant viruses carrying NPs with mutations affecting their anti-IFN-I function in their natural hosts, the mouse, would help to determine whether the NP-mediated inhibition of IFN-I influences the outcome of infection, including viral virulence and the ability to establish persistence. These observations, together with the elucidation of the mechanisms underlying the IFN-I-counteracting activity of arenavirus NPs, could contribute to a better understanding of the pathogenesis and immunogenicity of arenavirus infections. Results obtained from such studies may uncover new insights about arenavirus virulence, lead to the development of better antiviral drugs, and suggest new avenues for the generation of highly attenuated arenaviruses that could be considered as vaccine candidates.

## Abbreviations

2-5OAS: 2′-5′-oligoadenylate synthetase
ADAR: ds-RNA-specific adenosine deaminase
αDG: alpha dystroglycan
APOBEC: apolipoprotein B mRNA editing enzyme, catalytic polypeptide-like
ATF: activating transcription factor
BVDV: bovine viral diarrhea virus
CARD: caspase recruitment domain
CARDIF: CARD adaptor-inducing IFNβ
cDC: conventional DC
cDNA: complementary DNA
CpG: cytidine-phosphate-guanosine
DC: dendritic cell
ds: double-stranded
ER: endoplasmic reticulum
GPC: glycoprotein precursor
GTOV: Guanarito virus
HBV: hepatitis B virus
HCV: hepatitis C virus
HF: hemorrhagic fever
HIV: human immunodeficiency virus
HTLV-1: human T cell leukemia virus type 1
IRAK: IL-1 receptor-associated kinase
IFN: interferon
IFN-I: type I IFN
IFNAR: IFNα/β receptor
IGR: intergenic region
IL: interleukin
IKKα: IκB kinase α
IKKi: IκB kinase i
IPS-1: IFNβ promoter stimulator 1
IRF: interferon regulatory factor
ISG: IFN-stimulated gene
ISGF: ISG factor
ISRE: IFN-stimulated response element
JAK: Janus kinase
JUNV: Junin virus
LASV: Lassa virus
LCMV: lymphocytic choriomeningitis virus
LF: Lassa fever
LPS: lipopolysaccharide
M: matrix
MACV: Machupo virus
MAPK: mitogen-activated protein kinase
MAVS: mitochondrial antiviral signaling protein
MHC: major histocompatibility complex
MM: metallophilic macrophage
MZM: marginal zone macrophage
NDV: Newcastle disease virus
NF: nuclear factor
NK: natural killer
NLR: Nod-like receptor
NP: nucleoprotein
NS: negative stranded
NW: New World
OW: Old World
PAMP: pathogen-associated molecular pattern
pDC: plasmacytoid DC
PKR: protein kinase R
polyI:C: polyinosinic-polycytidylic acid
PRR: pattern-recognition receptor
RdRp: RNA-dependent RNA polymerase
RIG-I: retinoic acid-inducible gene 1
RING: really interesting new gene
RLR: RIG-I-like receptor
RNase L: ribonuclease L
RNP: ribonucleoprotein
SABV: Sabia virus
S1P: cellular site 1 protease
SKI-1: subtilisin-kexin-isozyme-1
ss: single-stranded
STAT: signal transducer and activator of transcription
TBK1: TANK-binding kinase 1
TCRV: Tacaribe virus
TIR: Toll/IL-1 receptor
TLR: Toll-like receptor
TNF: tumor necrosis factor
TRADD: TNF receptor short form 1A (TNFRSF1A)-associated via death domain
TRAF: TNF receptor-associated factor
TRAIL: TNF-related apoptosis-inducing ligand
TRIF: TIR-domain-containing adapter-inducing IFN-β
TRIM: tripartite motif
Tyk: tyrosine kinase
VISA: virus-induced signaling adaptor
VSV: vesicular stomatitis virus.
